# Perfluorodecanoic acid (PFDA) promotes gastric cell proliferation via sPLA2-IIA

**DOI:** 10.18632/oncotarget.17284

**Published:** 2017-04-20

**Authors:** Tianyi Dong, Yanping Peng, Ning Zhong, Fengyan Liu, Hanyu Zhang, Mengchen Xu, Rutao Liu, Mingyong Han, Xingsong Tian, Jihui Jia, Lap Kam Chang, Liang-Hong Guo, Shili Liu

**Affiliations:** ^1^ School of Medicine, Shandong University, Jinan, Shandong, 250012, China; ^2^ Department of Breast Thyroid Surgery, Shandong Provincial Hospital, Shandong University, Jinan, Shandong, 250021, China; ^3^ Department of Gastroenterology, Qilu Hospital of Shandong University, Jinan, Shandong, 250012, China; ^4^ School of Environmental Science and Engineering, Shandong University, Jinan, Shandong, 250100, China; ^5^ Cancer Therapy and Research Center, Shandong Provincial Hospital, Shandong university, Jinan, Shandong 250021, China; ^6^ State Key Laboratory of Environmental Chemistry and Ecotoxicology, Research Center for Eco-Environmental Sciences, Chinese Academy of Sciences, Beijing 100085, China

**Keywords:** PFDA, proliferation, sPLA2-IIA, TCF4

## Abstract

The association of perfluorodecanoicacid (PFDA) with tumor promotion and associated effects is not clear. Given that PDFA is mostly consumed with food and drinking water, we evaluated the effects of PFDA on a gastric cell line. When added to cell cultures, PFDA significantly increased growth rate and colony forming ability compared with control treatment. We found that suppression of cell senescence, but not apoptosis or autophagy was associated with PFDA-induced promotion of cell amount. To determine the molecular mechanism that was involved, DNA microarray assays was used to analyze changes in gene expression in response to PFDA treatment. Data analysis demonstrated that the vascular endothelial growth factor signaling pathway had the lowest *p*-value, with sPLA2-IIA (*pla2g2a*) exhibits the most altered expression pattern within the pathway. Moreover, sPLA2-IIA and its transcription factor TCF4, known as a direct target and a binding partner of Wnt/β-catenin signaling in gastric cells respectively, were the third and second most varied genes globally. Cells transfected with expression plasmids pENTER-*tcf4* and pENTER-*pla2g2a* show reduced cell proliferation by more than 60% and 30% respectively. Knockdown with sPLA2-IIA siRNA provided additional evidence that sPLA2-IIA was a mediator of PFDA-induced cell senescence suppression. The results suggest for the first time that PFDA induced suppression of cell senescence through inhibition of sPLA2-IIA protein expression and might increased the proliferative capacity of an existing tumor.

## INTRODUCTION

Perfluorinated carboxylic acids or perfluorinated fatty acids (PFCAs) have been used for decades to make products that resist heat, oil, and water. Because they are used in the manufacture of nonstick cookware, fire-fighting foam, and many other industrial products [1, 2], perfluorinated compounds can be detected globally in the environment [3, 4], wildlife [5–8] and humans [9–15]. In recent years, perfluorochemicals have become recognized as a public health concern as evidence of environmental persistence has increased [16–19], and toxicity data has emerged in laboratory animals [20–25].

Perfluorodecanoicacid (PFDA) is a perfluorinated carboxylic acid that is known to increase peroxisome proliferation in rodents by inducing various peroxisomal enzymes [26–28] as well as a series of mitochondrial, microsomal and cytosolic enzymes and proteins involved in lipid metabolism [29–32]. *In vivo*, PFDA is a highly potent and persistent peroxisome proliferator [33]. It is several times as toxic as perfluorooctanoic acid (PFOA) [34]. PFDA produces toxic effects similar to those caused by dioxin (2, 3, 7, 8-tetrachlorodibenzolydioxin), and has been reported to produce hypophagia and severe weightloss, bradycardia, hypothermia, and decreased serum thyroid hormone levels in rats [34, 35]. The reported cellular and physiological effects of PFDA include reproductive [36–39], endocrine [35, 40–42], and liver toxicity and disturbances of lipid metabolism [30, 43–47], and the immune system [48]. Evidence of damage to genetic material included DNA strand breaks and fragmentation, chromosomal breaks, and apoptosis [49, 50]. PFDA accumulates at much higher concentrations than PFOA in human blood and organs and serum elimination half-life can last several years. However, despite the evidence of PFDA toxicity, little is known of how it acts in tumor promotion.

Gastric cancer is a common malignancy, and accounts for about 10% of all invasive cancers worldwide. It may be the second leading cause of cancer death, and in China, the total number of cases and deaths from gastric cancer have increased concomitant with extensive demographic changes and ongoing increase of environmental pollution. A positive correlation of gastric cancer with environmental pollution has been confirmed [51–53]. PFDA is present in air, food, and water, especially in China, where 0.139 ng/mL PFDA was detected in snow fall in the area around Beijing [4]. In China, the primary source of PFDA accumulated in human is primarily through the polluted drinking water and food source especially the seafood [54]. In this study, we evaluated the effects of PFDA in gastric cells.

In the current study, our results suggested sPLA2-IIA mediated suppression of PFDA-induced cell senescence and then stimulated cell proliferation. This report describes a new molecular mechanism by which PFDA promotes cell growth in gastric cells.

## RESULTS

### Effects of PFDA on cell amount

**Figure 1:**
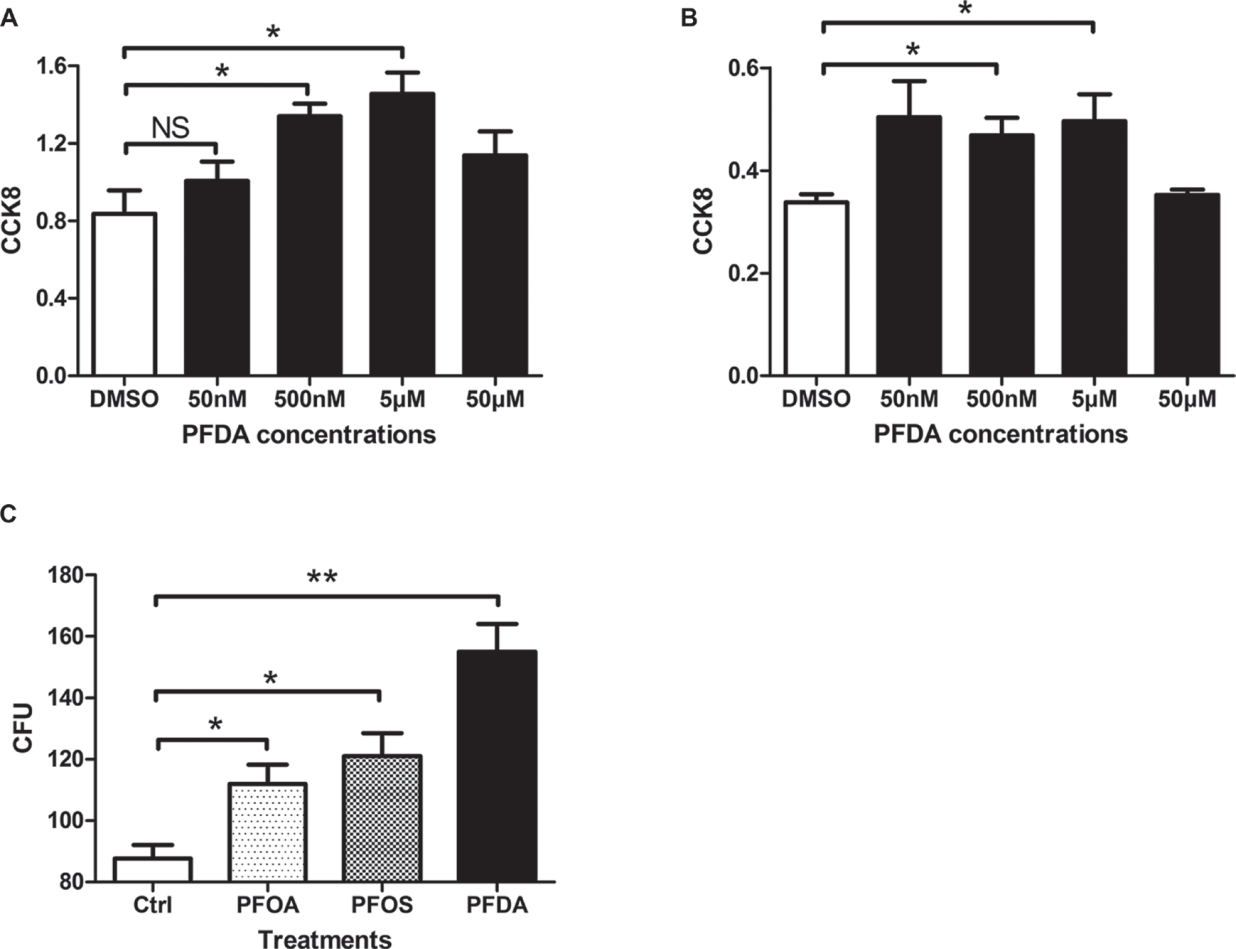
PFDA significantly enhanced cell amount (**A**) Cell counting kit 8 assay of PFDA treated or control AGS cells (*p < 0.05); (**B**) Cell counting kit 8 assay of Bel7402 treated with different concentrations of PFDA (*p < 0.05); (**C**) Quantification of colonies on agar. Colony formation assays were performed as described in Materials and Methods. Experiments were performed in triplicate with similar results.

To assess effects on *in vitro* cell proliferation, we treated AGS gastric epithelial cells with PFDA and monitored growth with a Cell Counting Kit-8 (CCK8) and a colony forming assay. As shown in Figure 1A, the CCK8 assays found that cells incubated with certain concentration of PFDA had significantly increased cell amount compared with DMSO-treated control cells. Moreover, the growth response of AGS cells varied in response to stimulation by different PFDA concentrations, this cell amount-promotion effect was verified by hepatic cell line Bel-7402 (Figure 1B) and another gastric cell line BGC823 (Supplementary Figure 1). More evidence was obtained from colony forming assay of AGS, PFDA enhanced colony forming ability by more than 70% compared with control cells (Supplementary Figure 2), which was significantly higher than that seen with PFOA, perfluorooctane sulfonate (PFOS) or other PFCs with longer chain length at the same concentration (PFOA and PFOS, 8C; PFDA, 10C; PFUDA, 11C; PFDoA, 12C; PFTeDA, 14C) (Figure 1C). The largest difference in growth rates was found on day 3 (Supplementary Figure 3). The results thus confirm that PFDA had an effect on the growth of human cells.

### PFDA enhanced gastric epithelial cells via suppressing senescence

**Figure 2:**
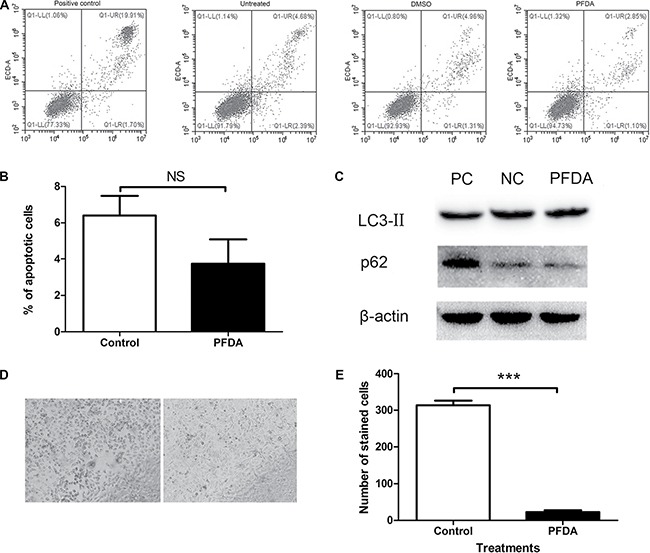
PFDA treatment suppressed senescence of gastric epithelial cells (**A**) Both PFDA treated and non-treated cells showed low levels of apoptosis, and their difference was not significant; (**B**) Quantification of the apoptotic cells, there was no significant difference between PFDA treated and control cells; (**C**) Western blot of LC3-II and p62. NC: DMSO, PC: starvation; (**D**) Senescence-associated β-galactosidase (SA-β-gal) activity assessment of PFDA treated and control cells; (**E**) Quantification of (D). ***p < 0.001. Apoptosis analysis, western blot and SA-β-gal activity assessment were performed as described in Materials and Methods.

Due to cell amount is generally affected by certain cellular processes such as apoptosis, autophagy and senescence, we used flow cytometry, western blots, and SA-β-gal staining to determine which cellular process were modulated by PFDA treatment. Following treatment with PFDA for 72 h, flow cytometry showed no significant difference between in the percentages of apoptotic cells with (3.7%) or without (6.4%) the presence of PFDA in the culture media (Figure 2A and 2B). Furthermore, the western blot data showed no differences in degradation of autophagy substrates (p62) or lipidation of LC3 (LC3-II) in response to PFDA treatment compared with controls (Figure 2C and Supplementary Figure 4). These results suggested that neither apoptosis nor autophagy were key factors in the PFDA-induced cell amount promotion. However, cell senescence-associated β-galactosidase (SA-β-gal) activity decreased following PFDA treatment, which was confirmed by a reduction in both the number of SA-β-gal-stained cells and in the staining intensity (Figure 2D and 2E). In addition, the decreased expression of p16, p21 and p27 as well as changes in cell morphology were consistent with a negative effect of PFDA on cell senescence (Supplementary Figure 5 and Supplementary Figure 6). Overall, these results implied that cell senescence played an important role in PFDA-induced promotion of AGS cell growth.

### sPLA2-IIA and its transcription factor TCF4, are down-regulated in PFDA-treated gastric epithelial cells

**Figure 3:**
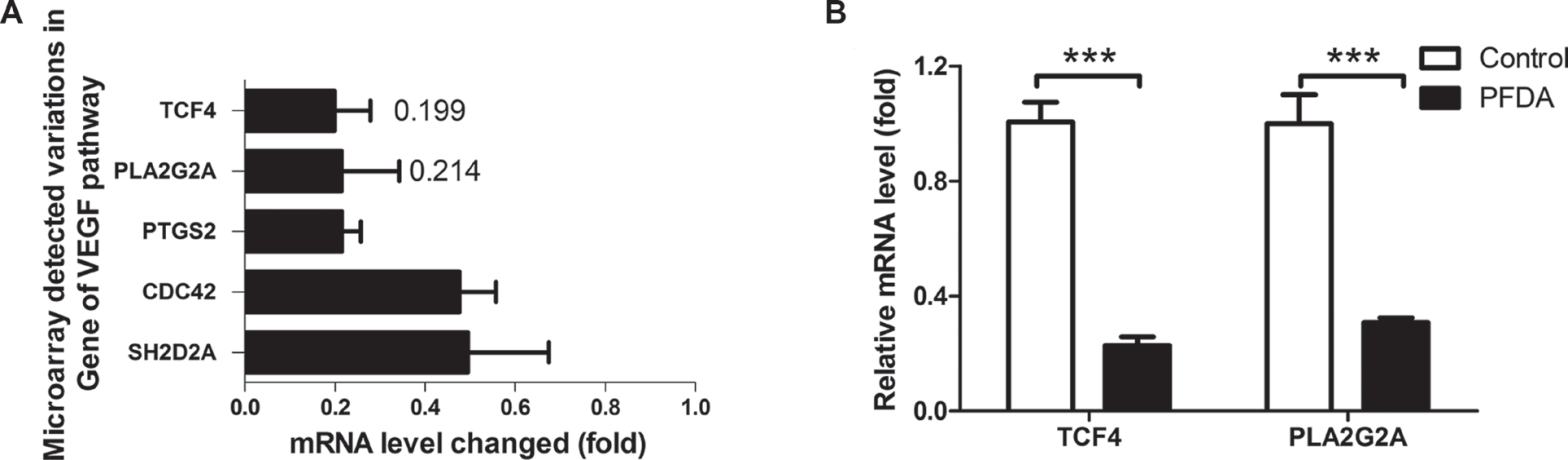
PFDA treatment down-regulated expression of sPLA2-IIA and its up-stream target gene TCF4 (**A**) Microarray analysis showed that TCF4 and PLA2G2A expression decreased 5.02 and 4.67 fold respectively concurrent with PFDA incubation; this decreased expression was verified by (**B**) RT-qPCR, ***p < 0.001. Microarray analysis and RT-qPCR were performed as described in the Materials and Methods.

**Figure 4:**
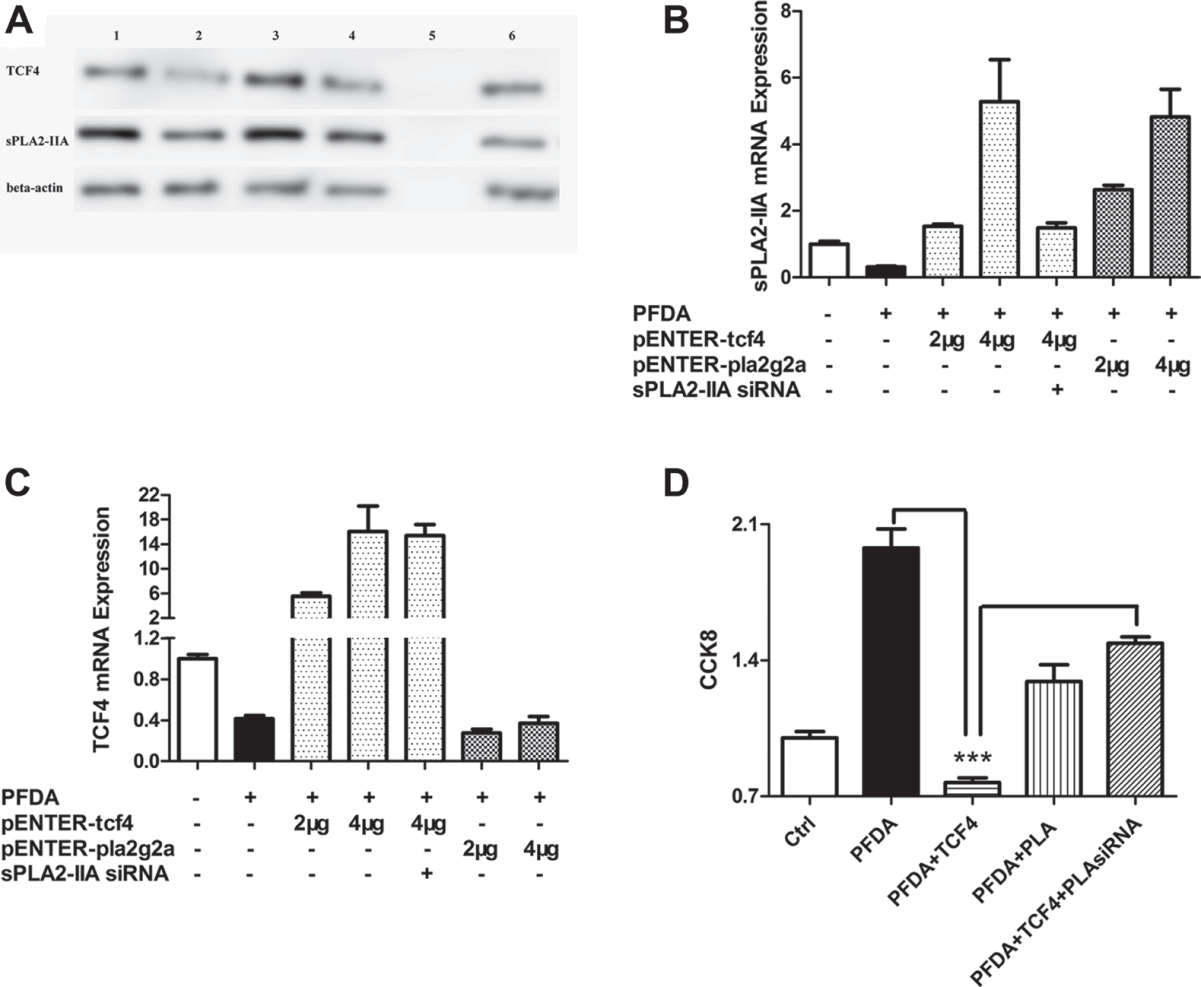
TCF4 and sPLA2-IIA were involved in PFDA-associated cell proliferation promotion (**A**) Western blot analysis of TCF4 and sPLA2-IIA expression in AGS cells: 1, DMSO control; 2, AGS with PFDA treatment; 3, AGS with PFDA treatment and pENTER-tcf4 transfection; 4, AGS with PFDA treatment and pENTER-pla2g2a transfection; 5, protein marker; 6, AGS with PFDA treatment, pENTER-tcf4 transfection and interfered by sPLA2-IIA siRNA; (**B**) and (**C**) RT-qPCR analysis of sPLA2-IIA and TCF4 expression in AGS cells under the circumstance of sPLA2-IIA and TCF4 plasmid transfection; (**D**) CCK8 analysis of AGS cells as TCF4 and sPLA2-IIA proteins were administrated, ***p < 0.001. Western blot and RT-qPCR were performed as described in Materials and Methods.

In the result of DAVID analysis of the microarray data, the biological process (GOTERM_BP_FAT) that was most affected was GO: 0014070, i.e., response to an organic substance (*p* = 0.00074, the smallest GOTERM *p*-value). In line with cellular response to PFDA treatment, The Kyoto Encyclopedia of Genes and Genomes (KEGG) pathway with the lowest *p*-value (*p* = 0.011) was the vascular endothelial growth factor (VEGF) signaling pathway. In that pathway, all four genes (CDC42, SH2D2A, PTGS2 and PLA2G2A) were down-regulated, and the most down-regulated gene was sPLA2-IIA (PLA2G2A). Its expression was 21.4% of that in controls, and it was the third most changed among all the genes analyzed (Figure 3A). The upstream transcription factor of sPLA2-IIA, TCF4, was the second most changed gene, with a decreased in expression to 19.9% of controls after PFDA treatment. The decreased expression was verified by RT-qPCR and in western blots (Figures 3B, 4A and Supplementary Figure 7). The down-regulation of sPLA2-IIA and TCF4 expression was also observed in BGC823 and Bel-7402 cells. sPLA2-IIA and TCF4 mRNAs decreased 5.88 and 5.29 fold respectively in BGC823 (Supplementary Figure 8) after PFDA treatments, they even decayed to undetected levels in Bel-7402 cells.

### TCF4 regulation of gastric cell growth by sPLA2-IIA is influenced by PFDA treatment

To investigate the effects of TCF4 and sPLA2-IIA gene expression on cell proliferation, we transfected AGS gastric epithelial cells with TCF4 and sPLA2-IIA expression plasmids (pENTER-*tcf4* and pENTER-*pla2g2a*) to stimulate TCF4 and sPLA2-IIA expression concomitant with PFDA treatment. Transfection was confirmed by sequencing using BLAST (Supplementary Figure 9). As shown in Figure 4A–4C, TCF4 and sPLA2-IIA expression was restored and expression levels of each gene were increased in dose-dependent manner after transfection. However, transfection of pENTER-*pla2g2a* did not affect TCF4 expression.

AGS cells were evaluated for changes in cell growth following transfection with pENTER-*tcf4* and pENTER-*pla2g2a*. As shown in Figure 4D, the growth rate of the transfected cells was reduced by 60% by pENTER-*tcf4* and 30% by pENTER-pla2g2a compared with PFDA-treated control cells. This is in line with the pattern of TCF4 and sPLA2-IIA expression in AGS cells, and was verified by CCK8 result of AGS transected with sPLA2-IIA a siRNA alone (Supplementary Figure 10). However, proliferation rates of cells transfected with pENTER-*tcf4* and -sPLA2-IIAsiRNA were higher than in cells with only pENTER-*tcf4* transfection, suggesting that PFDA induced cell proliferation through regulation of sPLA2-IIA protein expression.

### sPLA2-IIA expression restored cell senescence and inhibited cell proliferation

**Figure 5:**
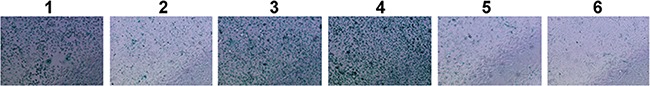
SA-β-gal activity assessment of PFDA treated and transfected AGS cells 1, DMSO control; 2, AGS with PFDA treatment; 3, AGS with PFDA treatment and pENTER-tcf4 transfection; 4, AGS with PFDA treatment and pENTER-pla2g2a transfection; 5, AGS with PFDA treatment, pENTER-tcf4 transfection and interfered by sPLA2-IIA siRNA; 6, AGS interfered by sPLA2-IIA siRNA. SA-β-gal activity assessment was performed as described in Materials and Methods.

The effects of sPLA2-IIA expression on cell senescence were investigated in AGS cells that over-expressed TCF4 and sPLA2-IIA. As shown in Figure 5, suppression of senescence was significantly increased two fold in cells expressing TCF4 and sPLA2-IIA compared with treatment-control cells. The results suggest that PFDA induced suppression of cell senescence and then stimulated cell proliferation through regulation of sPLA2-IIA protein expression.

## DISCUSSION

Despite recent advances in understanding the molecular mechanisms perfluorinated environmental pollutants, many unanswered questions remain. PFDA may important for understanding the molecular mechanisms of peroxisome proliferation. It is thus hoped that PFDA can assist in dissecting the sequence of events that is initiated by peroxisome proliferator-activated receptor interactions and ultimately results in tumor formation [33].

Of the phospholipase A2 super family (sPLA2) subgroup members, type-IIA sPLA2 (sPLA2-IIA, encoded by pla2g2a) have the highest bactericidal activities, and may also be involved in cell signaling, apoptosis, remodeling of cell membranes, and inflammatory responses [55–59]. Gastric cancer patients with tumors expressing high levels of sPLA2-IIA have been shown to have significantly improved survival compared with patients having tumors with low sPLA2-IIA expression [60]. However, beyond this prognostic association, little is actually known of how sPLA2-IIA contributes to development and progression of gastric cancer. Although sPLA2-IIA has been proposed as a potential tumor suppressor, evidence supporting this model is conflicting [60–64]. In the microarray data of AGS treated by PFDA, the KEGG pathways sPLA2-IIA belonging to were VEGF and MAPK signaling pathway. FOS and FGF18 were included in the latter pathway and increased by 2.28 and 2.14 fold respectively. Given that FOS and FGF18 are involved in tumor growth, and invasion as well as PFDA stimulates cell growth and, the microarray data seemed to imply us that PFDA were involved in the processes of tumorigenesis. However, a previous report found a larger group of genes differentially expressed in primary human hepatocytes, such as FABP1, Ehhadh, Pdk4, Scd1 etc. Meanwhile, FABP1 was also found up-regulated in our microarray data of AGS [65].

In early-stage tumors, Wnt signaling is active, and drives cell proliferation and dedifferentiation by up-regulating genes with pro-oncogenic activity. The transcriptional coactivator β-catenin can translocate to the nucleus to bind T-cell specific factor (TCF)-4 and lymphoid-enhancer factor (LEF)-1, thereby regulating gene expression [66]. Ganesan et al. showed that sPLA2-IIA is a direct target of Wnt/β-catenin signaling in gastric cancer cells and functions to negatively regulate gastric cancer invasiveness and metastasis [67]. In this study, the association of TCF4 and sPLA2-IIA expression was identified by gene over-expression and siRNA knockdown.

Cellular senescence is the state of permanent cell cycle arrest, and represents an important mechanism of both tumor suppression and tissue maintenance [68, 69]. Either oncogene inhibition or activation of tumor suppression can induce premature senescence, which then serves as a failsafe mechanism to restrict tumor development. On the other hand, recent reports showed that senescent cells enhanced cell malignancy. Senescent cells develop a secretory phenotype (SASP) that can affect the behavior of neighboring cells. Strikingly, many SASP factors are known to stimulate phenotypes associated with aggressive cancer cells, such as IL-6 and VEGF [70]. Senescence can be regulated by multiple pathways [71, 72]; however, the effects of PFDA and sPLA2-IIA on cellular senescence and its mechanism has not been reported previously. In this study, we demonstrated that PFDA enhanced growth of gastric epithelial cells. As to cell amount decreased after 24 and 48 h of PFDA treatment, the underlying mechanism could be PFDA suppressed glucose transport and NF-κB activation which directly affected the expression of a large number of genes and cell growth [73, 74]. The inhibiting the expression of sPLA2-IIA represents a new molecular mechanism engaged in regulating proliferation of gastric cancer cells.

## MATERIALS AND METHODS

Gastric adenocarcinoma cell line AGS and BGC823 were cultured and maintained in Ham's F-12 medium (HyClone, Utah, USA) supplemented with 10% FCS and 1% penicillin-streptomycin; the medium of Hepatocellular Carcinoma cell line Bel-7402 was RPMI-1640 (Life Technologies, California, USA) plus 10% FCS. The cultures were kept in a 5% CO2 and 95% air humidified incubator at 37°C. PFDA was purchased from Sigma Chemical Company (St. Louis, MO, USA). The full-length human sPLA2-IIA and TCF4 cDNA expression plasmids: pENTER-pla2g2a and pENTER-tcf4, and the control plasmid pENTER-mock were purchased from Biosune Company (Shanghai, China). FuGENEs HD Transfection Reagent (Roche Applied Science, Basel, Switzerland) was used for the transfection of these plasmids according to the manufacturer's instructions.

### RNA extraction and quantitative real-time PCR

Total cellular RNA was extracted with Trizol (Life Technologies, California, USA) according to the manufacturer's protocol. First-strand cDNA was synthesized from 1 μg of the extracted RNA using the RevertAid TM First Strand cDNA Synthesis Kit (Thermo Fisher Scientific, Massachusetts, USA). The cDNA was then amplified using specific primers as follow: human sPLA2-IIA forward primer 5′-TCACCCAAGAACTCTTACCA-3′ and reverse primer 5′-CAGCCGTAGAAGCCATAA-3′; human TCF4 forward primer 5′-GGGGCTCATACTCATCTTA-3′ and reverse primer 5′-CCCTATTGTAGTCGGCAGT-3′; β-actin forward primer 5′-AGTTGCGTTACACCCTTTCTTG-3′ and reverse primer 5′-CACCTTCACCGTTCCAGTTTT-3′. The real-time PCR reactions were performed on the ABI7000 Fast Real-Time PCR System with the SYBR Premix Ex Taq TM. The reaction ran for 35 cycles, in which each cycle included a denaturation step at 95°C for 10 sec, primer annealing step at 55°C for 30 sec and primer extension step at 72°C for 30 sec.

### Western blot analysis

Western Blot Analysis was performed as described previously [75]. Briefly, cell lysates (20 μg/lane) were separated on 10% SDS polyacrylamide gel and then were transferred to a poly (vinylidene fluoride) membrane. sPLA2-IIA, p62 and LC3-II protein [76] was detected by a mouse monoclonal IgG (OriGene Co. Ltd, Beijing, China) and visualized by the enhanced chemiluminescence system (Amersham, Arlington Heights, IL, USA). The intensity of the bands was quantitated using the NIH image software package. The extent of sPLA2-IIA expression was evaluated through the ratio of their expression in experiment groups over their corresponding expression in the control groups. If the ratio equated to more than 1, it may indicate its over-expression.

### Colony formation assay

Gastric cell line AGS was cultured in a 6-well plate at a concentration of 2 × 10^5^ cells/well. It was treated with PFDA (Sigma Chemical Company, St. Louis, MO, USA) and its control reagent solvent DMSO. After a period of growth, the cells were trypsinized and re-plated into new 6-well plates at a concentration of 300 cells/well. After making up the volume to 3 ml/ well with culture medium, the plates were incubated at 37°C for a week. During this time, the formation of cell colonies could become visible. At that point, the colonies were washed with PBS buffer for 3 times before subjected to cell fixation using 1 ml of methanol at room temperature for 10 min. Then, 1 ml of diluted Giemsa dye was added into each well and incubated at room temperature for about 20–25 min. After incubation, the wells were washed gently and repeatedly with PBS until no residual background Giemsa dye was observed. Finally, the 6-well plate was scanned for colony counting and analysis.

### Cell apoptosis analysis

Cell apoptosis analysis was performed as instructed by the Annexin V, FITC conjugate [77, 78] manual (Thermo Fisher Scientific). Briefly, 5 × 10^5^ AGS cells in a well of 6-well plates were treated with certain concentration of PFDA and control DMSO, incubating for 72 h before the cells were digested and harvested by centrifugation. After harvested the cells and washed in cold phosphate-buffered saline (PBS), then the cells were centrifuged and discard the supernatants, then re-suspended the cells in Annexin-binding buffer. Add 5 μl Annexin V-FITC conjugate and PI per 100 μl solution to the suspended cells. After 15 min at room temperature in the dark, 400 μl Annexin-binding buffer was added. Keep the cells on ice and analyzed with flow cytometry equipped with an argon laser at 488 nm.

### Senescence-associated β-galactosidase (SA-β-gal) activity assessment

SA-β-gal activity was detected using a staining kit of SA-β-gal [79, 80], and it was performed according to the manufacturer's protocol. Briefly, AGS cells were seeded into a 24-well plate. The cells were treated with PFDA on day 3. After treatment, the cells were washed with PBS (pH7.2) twice before they were fixed with 3.7% formaldehyde in PBS for 3–5 min. Then, the SA-β-gal staining solution (1 mg/ml X-gal, 40 mM citric acid/sodium phosphate pH6.0, 5 mM potassium ferrocyanide, 150 mM NaCl, and 2 mM MgCl_2_) was added into each well before the plate was incubated at 37°C for 12–16 hr in the absence of CO_2_. Finally, the cells were rinsed with PBS and the plate was observed under the microscope, in which the number of the SA-β-gal positive cells was recorded. The experiment was performed in triplicate.

### Microarray analysis

The microarray chip consisted of 27326 probes for different human cDNAs (Capitalbio Company, Beijing, China), in which the house-keeping gene glyceraldehyde-3-phosphate dehydrogenase (GAPDH) was served as internal control. The cDNAs extracted from PFDA-treated AGS cells were labeled with Cy3, while the cDNAs from the control DMSO-treated AGS cells were labeled with Cy5. The labeled cDNAs were then hybridized with microarray chip under standard conditions according to manufacturer's instructions. Changes in mRNA expression in response to PFDA stimulation were assayed in DNA microarrays. Two fold up- or down regulation were set as cutoff values, and changes in gene expression were analyzed using the Database for Annotation, Visualization and Integrated Discovery (DAVID).

### Statistical data analysis

All experiments except microarray analysis were repeated at least three times and the data were expressed as mean ± standard deviation (SD). The differences between the three groups were compared using the Student's *t*-tests and *P* < 0.05 (*) was considered statistically significant.

## SUPPLEMENTARY MATERIALS FIGURES


