# Supplementary material for: Perfluorodecanoic acid (PFDA) promotes gastric cell proliferation via sPLA2-IIA
Source: Oncotarget. 2017 Apr 20;8(31):50911–20. doi: 10.18632/oncotarget.17284 (PMC5584216; doi:10.18632/oncotarget.17284)
Supplement: Supplementary file 1 [file oncotarget-08-50911-s001.pdf]

# Perfluorodecanoic acid (PFDA) promotes gastric cell proliferation via sPLA2-IIA

## Supplementary Materials

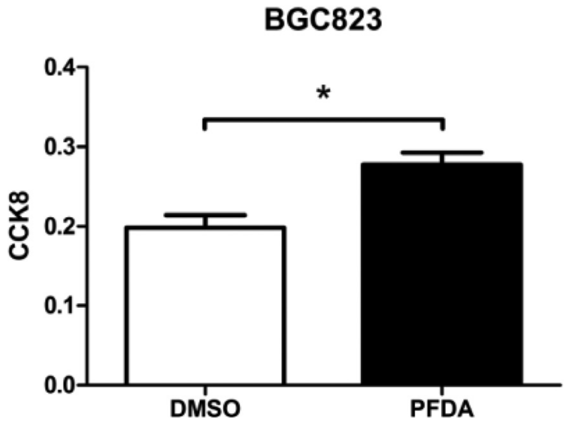


**Supplementary Figure 1: Cell counting kit 8 assay of PFDA or DMSO treated cells.** Cells incubated with PFDA had significantly increased cell amount compared with DMSO-treated control cells. Experiments were performed in triplicate with similar results.

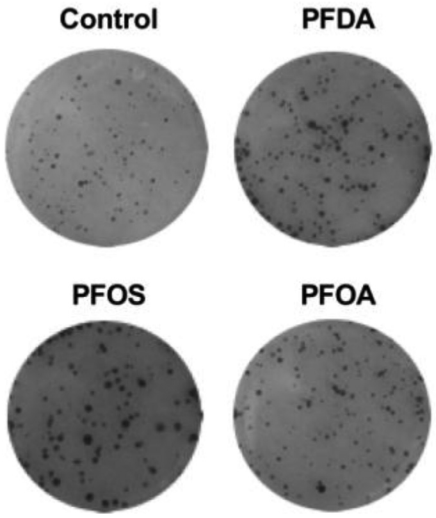


**Supplementary Figure 2: Colonies formed on agar.** Colony formation assay were performed as described in Materials and Methods Experiments were performed in triplicate with similar results.

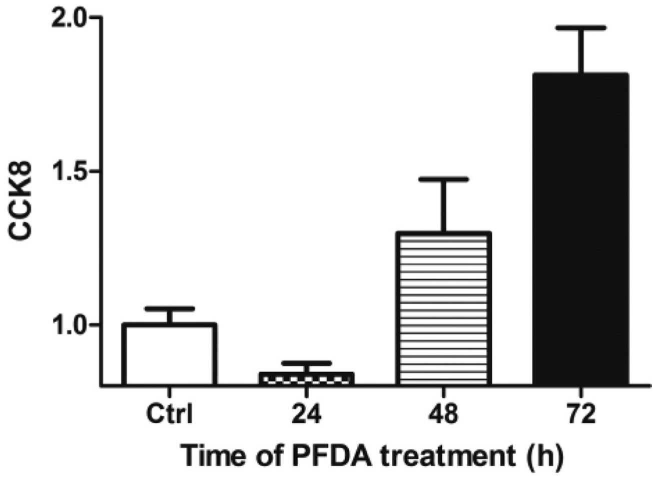


**Supplementary Figure 3: AGS cells proliferation in response to PFDA stimulated at different time.** Cell counting kit 8 assays were performed as described in Kit instruction. Experiments were performed in triplicate with similar results.

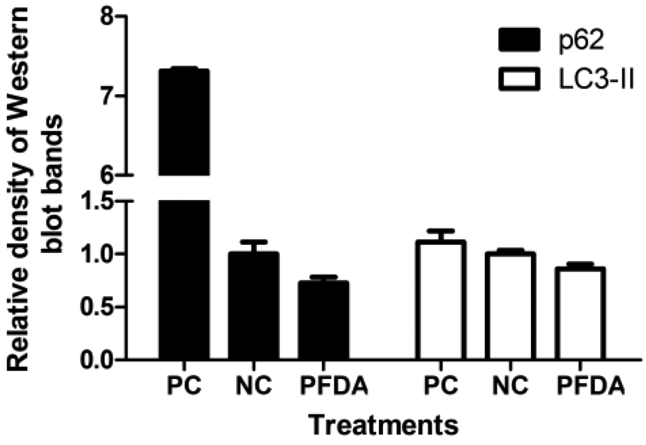


**Supplementary Figure 4: Quantification of western blot bands, the average density of NC in both experiments was defined as 1.** Experiments were performed in triplicate with similar results.

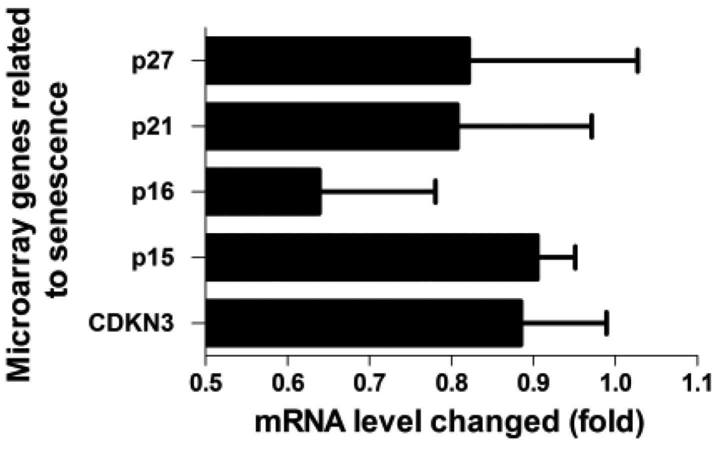


**Supplementary Figure 5: Microarray analysis showed some genes related to senescence decreased their expression concurrent with PFDA incubation.**

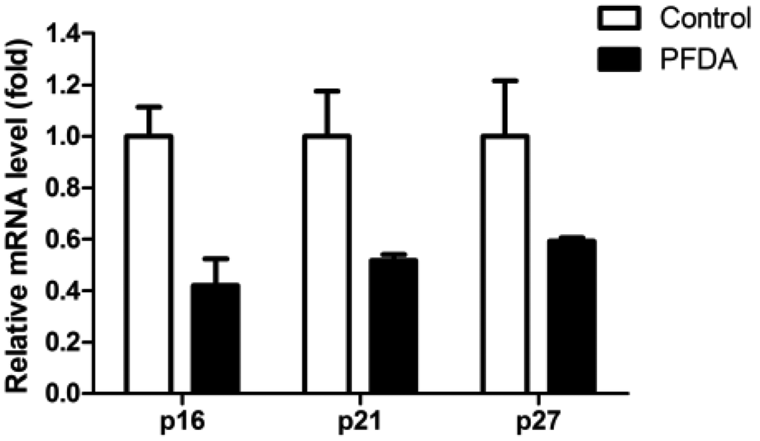


**Supplementary Figure 6: QRT-PCR results of p16, p21, and p27 mRNA in AGS showed that PFDA treatment down-regulated expression of p16, p21, and p27.**

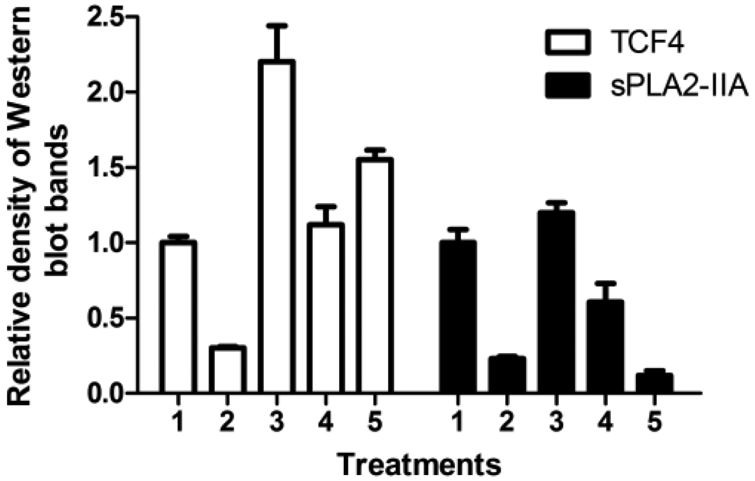


**Supplementary Figure 7: Quantification of western blot bands.** 1, DMSO control; 2, AGS with PFDA treatment; 3, AGS with PFDA treatment and pENTER-tcf4 transfection; 4, AGS with PFDA treatment and pENTER-pla2g2a transfection; 5, AGS with PFDA treatment, pENTER-tcf4 transfection and interfered by sPLA2-IIA siRNA.

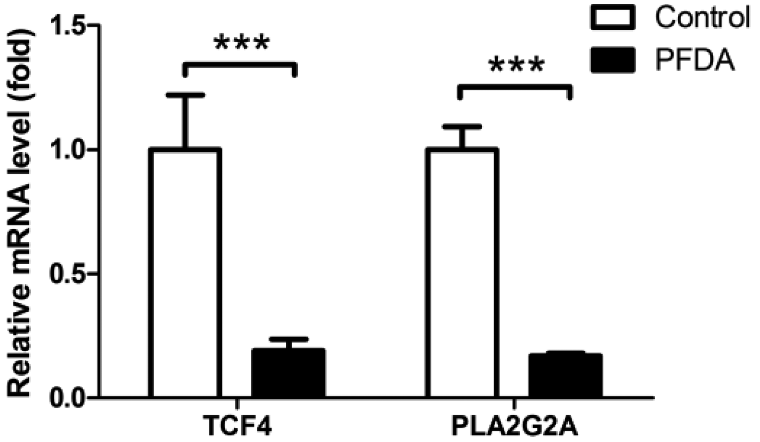


**Supplementary Figure 8: QRT-PCR results of sPLA2-IIA and TCF4 in BGC823 showed that PFDA treatment down-regulated expression of sPLA2-IIA and TCF4.** Experiments were performed in triplicate with similar results.

G03_J20638_J44931a_TCF4_5'_10P

GAGTTCGTACACTCCGCCCATTGACGCAATGGGCGGTAGGCGTGTACGGTGGGAGGTCTATATAAGCAGAGCTGGTTTAG

TGAACCGTCAGATCCGCTAGTAATACGACTCACTATAGGGAGAGGATCCGGTACCGAGGAGATCTGCCGCCGCGATCGCC

ATGCATCACCAACAGCGAATGGCTGCCTTAGGGACGGACAAAGAGCTGAGTGATTTACTGGATTTCAGTGCGATGTTTTC

ACCTCCTGTGAGCAGTGGGAAAAATGGACCAACTTCTTTGGCAAGTGGACATTTTACTGGCTCAAATGTAGAAGACAGAA

GTAGCTCAGGGTCCTGGGGGAATGGAGGACATCCAAGCCCGTCCAGGAACTATGGAGATGGGACTCCCTATGACCACATG

ACCAGCAGGGACCTTGGGTCACATGACAATCTCTCTCCACCTTTTGTCAATTCCAGAATACAAAGTAAAACAGAAAGGGG

CTCATACTCATCTTATGGGAGAGAATCAAACTTACAGGGTTGCCACCAGCAGAGTCTCCTTGGAGGTGACATGGATATGG

GCAACCCAGGAACCCTTTCGCCCACCAAACCTGGTTCCCAGTACTATCAGTATTCTAGCAATAATCCCCGAAGGAGGCCT

CTTCACAGTAGTGCCATGGAGGTACAGACAAAGAAAGTTCGAAAAGTTCCTCCAGGTTTGCCATCTTCAGTCTATGCTCC

ATCAGCAAGCACTGCCGACTACAATAGGGACTCGCCAGGCTATCCTTCCTCCAAACCAGCAACCAGCACTTTCCCTAGCT

CCTTCTTCATGCAAGATGGCCATCACAGCAGTGACCCTTGGAGCTCCTCCAGTGGGATGAATCAGCCTGGCTATGCAGGA

ATGTTGGGCAACTCTTCTCATATTCCACAGTCCAGCAGCTACTGTAGCCTGCATCCACATGAACGTTTGAGCTATCCATC

ACACTCCTCAGCAGACATCAATTTCCAGTCTTCCTCCGAT

PREDICTED: Homo sapiens transcription factor 4 (TCF4), transcript variant X7, mRNA

Sequence ID: ref|XM_006722537.2|Length: 6767Number of Matches: 1 Related Information

Gene-associated gene details

Range 1: 169 to 1007GenBankGraphics Next Match Previous Match

Alignment statistics for match #1

| Score | Expect | Identities | Gaps | Strand |
|---|---|---|---|---|
| 1544 bits(836) | 0.0 | 839/840(99%) | 1/840(0%) | Plus/Plus |

Query 161 ATGCATCACCAACAGCGAATGGCTGCCTTAGGGACGGACAAAGAGCTGAGTGATTTACTG 220

||||||||||||||||||||||||||||||||||||||||||||||||||||||||||||

Sbjct 169 ATGCATCACCAACAGCGAATGGCTGCCTTAGGGACGGACAAAGAGCTGAGTGATTTACTG 228

Query 221 GATTTCAGTGCGATGTTTTCACCTCCTGTGAGCAGTGGGAAAAATGGACCAACTTCTTTG 280

||||||||||||||||||||||||||||||||||||||||||||||||||||||||||||

Sbjct 229 GATTTCAGTGCGATGTTTTCACCTCCTGTGAGCAGTGGGAAAAATGGACCAACTTCTTTG 288

Query 281 GCAAGTGGACATTTTACTGGCTCAAATGTAGAAGACAGAAGTAGCTCAGGGTCCTGGGGG 340

||||||||||||||||||||||||||||||||||||||||||||||||||||||||||||

Sbjct 289 GCAAGTGGACATTTTACTGGCTCAAATGTAGAAGACAGAAGTAGCTCAGGGTCCTGGGGG 348

```
Query  341   AATGGAGGACATCCAAGCCCGTCCAGGAACTATGGAGATGGGACTCCCTATGACCACATG  400
             ||||||||||||||||||||||||||||||||||||||||||||||||||||||||||||
Sbjct  349   AATGGAGGACATCCAAGCCCGTCCAGGAACTATGGAGATGGGACTCCCTATGACCACATG  408

Query  401   ACCAGCAGGGACCTTGGGTCACATGACAATCTCTCTCCACCTTTTGTCAATTCCAGAATA  460
             ||||||||||||||||||||||||||||||||||||||||||||||||||||||||||||
Sbjct  409   ACCAGCAGGGACCTTGGGTCACATGACAATCTCTCTCCACCTTTTGTCAATTCCAGAATA  468

Query  461   CAAAGTAAAACAGAAAGGGGCTCATACTCATCTTATGGGAGAGAATCAAACTTACAGGGT  520
             ||||||||||||||||||||||||||||||||||||||||||||||||||||||||||||
Sbjct  469   CAAAGTAAAACAGAAAGGGGCTCATACTCATCTTATGGGAGAGAATCAAACTTACAGGGT  528

Query  521   TGCCACCAGCAGAGTCTCCTTGGAGGTGACATGGATATGGGCAACCCAGGAACCCTTTCG  580
             ||||||||||||||||||||||||||||||||||||||||||||||||||||||||||||
Sbjct  529   TGCCACCAGCAGAGTCTCCTTGGAGGTGACATGGATATGGGCAACCCAGGAACCCTTTCG  588

Query  581   CCCACCAAACCTGGTTCCCAGTACTATCAGTATTCTAGCAATAATCCCCGAAGGAGGCCT  640
             ||||||||||||||||||||||||||||||||||||||||||||||||||||||||||||
Sbjct  589   CCCACCAAACCTGGTTCCCAGTACTATCAGTATTCTAGCAATAATCCCCGAAGGAGGCCT  648

Query  641   CTTCACAGTAGTGCCATGGAGGTACAGACAAAGAAAGTTCGAAAAGTTCCTCCAGGTTTG  700
             ||||||||||||||||||||||||||||||||||||||||||||||||||||||||||||
Sbjct  649   CTTCACAGTAGTGCCATGGAGGTACAGACAAAGAAAGTTCGAAAAGTTCCTCCAGGTTTG  708

Query  701   CCATCTTCAGTCTATGCTCCATCAGCAAGCACTGCCGACTACAATAGGGACTCGCCAGGC  760
             ||||||||||||||||||||||||||||||||||||||||||||||||||||||||||||
Sbjct  709   CCATCTTCAGTCTATGCTCCATCAGCAAGCACTGCCGACTACAATAGGGACTCGCCAGGC  768

Query  761   TATCCTTCCTCCAAACCAGCAACCAGCACTTTCCCTAGCTCCTTCTTCATGCAAGATGGC  820
             ||||||||||||||||||||||||||||||||||||||||||||||||||||||||||||
Sbjct  769   TATCCTTCCTCCAAACCAGCAACCAGCACTTTCCCTAGCTCCTTCTTCATGCAAGATGGC  828

Query  821   CATCACAGCAGTGACCCTTGGAGCTCCTCCAGTGGGATGAATCAGCCTGGCTATGCAGGA  880
             ||||||||||||||||||||||||||||||||||||||||||||||||||||||||||||
Sbjct  829   CATCACAGCAGTGACCCTTGGAGCTCCTCCAGTGGGATGAATCAGCCTGGCTATGCAGGA  888

Query  881   ATGTTGGGCAACTCTTCTCATATTCCACAGTCCAGCAGCTACTGTAGCCTGCATCCACAT  940
             ||||||||||||||||||||||||||||||||||||||||||||||||||||||||||||
Sbjct  889   ATGTTGGGCAACTCTTCTCATATTCCACAGTCCAGCAGCTACTGTAGCCTGCATCCACAT  948

Query  941   GAACGTTTGAGCTATCCATCACACTCCTCAGCAGACATCAATTTCCAGTCTTCCTCCGAT  1000
             ||||||||||||||||||||||||||||||||||||||||| ||||||||||||||||||
Sbjct  949   GAACGTTTGAGCTATCCATCACACTCCTCAGCAGACATCAA-TTCCAGTCTTCCTCCGAT  1007
```

G04_J20638_J44931b_TCF4_3'_10P

GCGTACGTTCTAGATCGGTGGACGGATATCTTATCTAGATCCGGTGGATCGGATAAACCTTATTAGTGGTGGTGGTGGTG

GTGCTCGACGAATTTATCGTCGTCATCCTTATAATCCTCGAGCGGCCGCGTACGCGTCATCTGTCCCATGTGATTCGATG

CGTCTCCCATTCCAGGGTGTGGGCCGGCCAAGGAGAGAGGGGGAGGCTCTGAGGACACCTTCTCTTCCTCCCTTCTTTTC

AGACACGCAGCTTTCGGATTCAGATTCCTTTCTCGGACTTGCTGCTCCAGACTGAGGATGACGGCCACCGCCTGGTGGAG

GATCAGGAGCTTGGTCTGGGGCTTGTCACTCTTGAGGTGGAGCTGCACCATGCGGCCGAGCTCTTTGAAAGCCTCGTTGA

TGTCACGGACCCGCAGACGCTCTCGGGCATTGTTGGCCATCCTCCGCTCCTTCTCACGCTCTGCCTTCTGCTCTGGTGTC

AGGTCCTCATCGTCATTATTGCTAGTAATTGATTTGATATCCTTCTTGTCGTCATCTAATTTCTTGTCCTCCGAAGATTT

CGTGTCTTGCAGGTTCTCATCACCCTCGTCATCGGATTTGATCTCAGAGCTGCCAGAGGAGACACTCTGCCCCTGTAGTC

CTGGTGGCATGCCTCTGTAAGGGTCCTGGGGTGGGTTCAGGTCAGGGGAAGTCGCAGACTGGACAGGAAGCTGTGGAACC

GGAACCTGGTTTGGCAGAAGAGAATGGCTGCCTCTCAGGGCCACGCCATCTTCACGATGGGTCCCCACCATGAGTGAATG

TCTGTTGGCTGAAAGAAGGCCGGTTCCATACCCTGAGCCCAGACCACCCATGGCTCCATTATGAGAAAGGTCCAATGATT

CCATGCATGTCCCCATGACCACCAGGCATAGCTGTGGATGGGCCCACTGCATGGTTCCGGAGAACATGAATAGCATCATC

CAGTCTTTCTAAACGATCTTCAATTC

PREDICTED: Homo sapiens transcription factor 4 (TCF4), transcript variant X28, mRNA

Sequence ID: ref|XM_006722540.2|Length: 6326Number of Matches: 1 Related Information

Gene-associated gene details

Range 1: 893 to 1740GenBankGraphics Next Match Previous Match

Alignment statistics for match #1

| Score | Expect | Identities | Gaps | Strand |
|---|---|---|---|---|
| 1561 bits(845) | 0.0 | 848/849(99%) | 1/849(0%) | Plus/Minus |

Query 138 CATCTGTCCCATGTGATTCGATGCGTCTCCCATTCCAGGGTGTGGGCCGGCCAAGGAGAG 197

||||||||||||||||||||||||||||||||||||||||||||||||||||||||||||

Sbjct 1740 CATCTGTCCCATGTGATTCGATGCGTCTCCCATTCCAGGGTGTGGGCCGGCCAAGGAGAG 1681

Query 198 AGGGGGAGGCTCTGAGGACACCTTCTCTTCCTCCCTTCTTTTCAGACACGCAGCTTTCGG 257

||||||||||||||||||||||||||||||||||||||||||||||||||||||||||||

Sbjct 1680 AGGGGGAGGCTCTGAGGACACCTTCTCTTCCTCCCTTCTTTTCAGACACGCAGCTTTCGG 1621

Query 258 ATTCAGATTCCTTTCTCGGACTTGCTGCTCCAGACTGAGGATGACGGCCACCGCCTGGTG 317

||||||||||||||||||||||||||||||||||||||||||||||||||||||||||||

Sbjct 1620 ATTCAGATTCCTTTCTCGGACTTGCTGCTCCAGACTGAGGATGACGGCCACCGCCTGGTG 1561

Query 318 GAGGATCAGGAGCTTGGTCTGGGGCTTGTCACTCTTGAGGTGGAGCTGCACCATGCGGCC 377

||||||||||||||||||||||||||||||||||||||||||||||||||||||||||||

Sbjct 1560 GAGGATCAGGAGCTTGGTCTGGGGCTTGTCACTCTTGAGGTGGAGCTGCACCATGCGGCC 1501

Query 378 GAGCTCTTTGAAAGCCTCGTTGATGTCACGGACCCGCAGACGCTCTCGGGCATTGTTGGC 437

||||||||||||||||||||||||||||||||||||||||||||||||||||||||||||

Sbjct 1500 GAGCTCTTTGAAAGCCTCGTTGATGTCACGGACCCGCAGACGCTCTCGGGCATTGTTGGC 1441

Query 438 CATCCTCCGCTCCTTCTCACGCTCTGCCTTCTGCTCTGGTGTCAGGTCCTCATCGTCATT 497

```
            ||||||||||||||||||||||||||||||||||||||||||||||||||||||||||||
Sbjct  1440  CATCCTCCGCTCCTTCTCACGCTCTGCCTTCTGCTCTGGTGTCAGGTCCTCATCGTCATT  1381

Query  498   ATTGCTAGTAATTGATTTGATATCCTTCTTGTCGTCATCTAATTTCTTGTCCTCCGAAGA  557
             ||||||||||||||||||||||||||||||||||||||||||||||||||||||||||||
Sbjct  1380  ATTGCTAGTAATTGATTTGATATCCTTCTTGTCGTCATCTAATTTCTTGTCCTCCGAAGA  1321

Query  558   TTTCGTGTCTTGCAGGTTCTCATCACCCTCGTCATCGGATTTGATCTCAGAGCTGCCAGA  617
             ||||||||||||||||||||||||||||||||||||||||||||||||||||||||||||
Sbjct  1320  TTTCGTGTCTTGCAGGTTCTCATCACCCTCGTCATCGGATTTGATCTCAGAGCTGCCAGA  1261

Query  618   GGAGACACTCTGCCCCTGTAGTCCTGGTGGCATGCCTCTGTAAGGGTCCTGGGGTGGGTT  677
             ||||||||||||||||||||||||||||||||||||||||||||||||||||||||||||
Sbjct  1260  GGAGACACTCTGCCCCTGTAGTCCTGGTGGCATGCCTCTGTAAGGGTCCTGGGGTGGGTT  1201

Query  678   CAGGTCAGGGGAAGTCGCAGACTGGACAGGAAGCTGTGGAACCGGAACCTGGTTTGGCAG  737
             ||||||||||||||||||||||||||||||||||||||||||||||||||||||||||||
Sbjct  1200  CAGGTCAGGGGAAGTCGCAGACTGGACAGGAAGCTGTGGAACCGGAACCTGGTTTGGCAG  1141

Query  738   AAGAGAATGGCTGCCTCTCAGGGCCACGCCATCTTCACGATGGGTCCCCACCATGAGTGA  797
             ||||||||||||||||||||||||||||||||||||||||||||||||||||||||||||
Sbjct  1140  AAGAGAATGGCTGCCTCTCAGGGCCACGCCATCTTCACGATGGGTCCCCACCATGAGTGA  1081

Query  798   ATGTCTGTTGGCTGAAAGAAGGCCGGTTCCATACCCTGAGCCCAGACCACCCATGGCTCC  857
             ||||||||||||||||||||||||||||||||||||||||||||||||||||||||||||
Sbjct  1080  ATGTCTGTTGGCTGAAAGAAGGCCGGTTCCATACCCTGAGCCCAGACCACCCATGGCTCC  1021

Query  858   ATTATGAGAAAGGTCCAATGATTCCATGCATGTCCCCATGACCACCAGGCATAGCTGTGG  917
             |||||||||| |||||||||||||||||||||||||||||||||||||||||||||||||
Sbjct  1020  ATTATGAGAA-GGTCCAATGATTCCATGCATGTCCCCATGACCACCAGGCATAGCTGTGG  962

Query  918   ATGGGCCCACTGCATGGTTCCGGAGAACATGAATAGCATCATCCAGTCTTTCTAAACGAT  977
             ||||||||||||||||||||||||||||||||||||||||||||||||||||||||||||
Sbjct  961   ATGGGCCCACTGCATGGTTCCGGAGAACATGAATAGCATCATCCAGTCTTTCTAAACGAT  902

Query  978   CTTCAATTC  986
             |||||||||
Sbjct  901   CTTCAATTC  893
```

(b)

G05_J20638_J44932a_PLA2G2A_5'

AAAGTTCGTACACTCCGCCCATTGACGCAATGGGCGGTAGGCGTGTACGGTGGGAGGTCTATATAAGCAGAGCTGGTTTA
GTGAACCGTCAGATCCGCTAGTAATACGACTCACTATAGGGAGAGGATCCGGTACCGAGGAGATCTGCCGCCGCGATCGC
CATGAAGACCCTCCTACTGTTGGCAGTGATCATGATCTTTGGCCTACTGCAGGCCCATGGGAATTTGGTGAATTTCCACA

GAATGATCAAGTTGACGACAGGAAAGGAAGCCGCACTCAGTTATGGCTTCTACGGCTGCCACTGTGGCGTGGGTGGCAGA

GGATCCCCCAAGGATGCAACGGATCGCTGCTGTGTCACTCATGACTGTTGCTACAAACGTCTGGAGAAACGTGGATGTGG

CACCAAATTTCTGAGCTACAAGTTTAGCAACTCGGGGAGCAGAATCACCTGTGCAAAACAGGACTCCTGCAGAAGTCAAC

TGTGTGAGTGTGATAAGGCTGCTGCCACCTGTTTTGCTAGAAACAAGACGACCTACAATAAAAAGTACCAGTACTATTCC

AATAAACACTGCAGAGGGAGCACCCCTCGTTGCACGCGTACGCGGCCGCTCGAGGATTATAAGGATGACGACGATAAATT

CGTCGAGCACCACCACCACCACCACTAATAAGGTTTATCCGATCCACCGGATCTAGATAAGATATCCGATCCACCGGATC

TAGATAACTGATCATAATCAGCCATACCACATTTGTAGAGGTTTTACTTGCTTTAAAAAACCTCCCACACCTCCCCCTGA

ACCTGAAACATAAAATGAATGCAATTGTTGTTGTTAACTTGTTTATTGCAGCTTATAATGGTTACAAATAAAGCAATAGC

ATCACAAATTTCACAAATAAAGCATTTTTTTCACTGCATTCTAGTTGTGGTTTGTCCAAACTCATCAATGTATCTTAACG

CGGATCTGGGCGTGGTTAAGGGTGGGAAAGAATA

Homo sapiens phospholipase A2, group IIA (platelets, synovial fluid) (PLA2G2A), transcript variant 4, mRNA

Sequence ID: ref|NM_001161729.1|Length: 940Number of Matches: 1

Related Information

Gene-associated gene details

GEO Profiles-microarray expression data

Map Viewer-aligned genomic context

Range 1: 217 to 650GenBankGraphics Next Match Previous Match

Alignment statistics for match #1

| Score | Expect | Identities | Gaps | Strand |
|---|---|---|---|---|
| 802 bits(434) | 0.0 | 434/434(100%) | 0/434(0%) | Plus/Plus |

Query 160 CCATGAAGACCCTCCTACTGTTGGCAGTGATCATGATCTTTGGCCTACTGCAGGCCCATG 219

||||||||||||||||||||||||||||||||||||||||||||||||||||||||||||

Sbjct 217 CCATGAAGACCCTCCTACTGTTGGCAGTGATCATGATCTTTGGCCTACTGCAGGCCCATG 276

Query 220 GGAATTTGGTGAATTTCCACAGAATGATCAAGTTGACGACAGGAAAGGAAGCCGCACTCA 279

||||||||||||||||||||||||||||||||||||||||||||||||||||||||||||

Sbjct 277 GGAATTTGGTGAATTTCCACAGAATGATCAAGTTGACGACAGGAAAGGAAGCCGCACTCA 336

Query 280 GTTATGGCTTCTACGGCTGCCACTGTGGCGTGGGTGGCAGAGGATCCCCCAAGGATGCAA 339

||||||||||||||||||||||||||||||||||||||||||||||||||||||||||||

Sbjct 337 GTTATGGCTTCTACGGCTGCCACTGTGGCGTGGGTGGCAGAGGATCCCCCAAGGATGCAA 396

Query 340 CGGATCGCTGCTGTGTCACTCATGACTGTTGCTACAAACGTCTGGAGAAACGTGGATGTG 399

||||||||||||||||||||||||||||||||||||||||||||||||||||||||||||

Sbjct 397 CGGATCGCTGCTGTGTCACTCATGACTGTTGCTACAAACGTCTGGAGAAACGTGGATGTG 456

Query 400 GCACCAAATTTCTGAGCTACAAGTTTAGCAACTCGGGGAGCAGAATCACCTGTGCAAAAC 459

||||||||||||||||||||||||||||||||||||||||||||||||||||||||||||

Sbjct 457 GCACCAAATTTCTGAGCTACAAGTTTAGCAACTCGGGGAGCAGAATCACCTGTGCAAAAC 516

Query 460 AGGACTCCTGCAGAAGTCAACTGTGTGAGTGTGATAAGGCTGCTGCCACCTGTTTTGCTA 519

||||||||||||||||||||||||||||||||||||||||||||||||||||||||||||

Sbjct 517 AGGACTCCTGCAGAAGTCAACTGTGTGAGTGTGATAAGGCTGCTGCCACCTGTTTTGCTA 576

Query 520 GAAACAAGACGACCTACAATAAAAAGTACCAGTACTATTCCAATAAACACTGCAGAGGGA 579

||||||||||||||||||||||||||||||||||||||||||||||||||||||||||||

Sbjct 577 GAAACAAGACGACCTACAATAAAAAGTACCAGTACTATTCCAATAAACACTGCAGAGGGA 636

Query 580 GCACCCCTCGTTGC 593

||||||||||||||

Sbjct 637 GCACCCCTCGTTGC 650

(c)

G06_J20638_J44932b_PLA2G2A_3'

AGGATACGTTCTAGATCGGTGGACGGATATCTTATCTAGATCCGGTGGATCGGATAAACCTTATTAGTGGTGGTGGTGGT

GGTGCTCGACGAATTTATCGTCGTCATCCTTATAATCCTCGAGCGGCCGCGTACGCGTGCAACGAGGGGTGCTCCCTCTG

CAGTGTTTATTGGAATAGTACTGGTACTTTTTATTGTAGGTCGTCTTGTTTCTAGCAAAACAGGTGGCAGCAGCCTTATC

ACACTCACACAGTTGACTTCTGCAGGAGTCCTGTTTTGCACAGGTGATTCTGCTCCCCGAGTTGCTAAACTTGTAGCTCA

GAAATTTGGTGCCACATCCACGTTTCTCCAGACGTTTGTAGCAACAGTCATGAGTGACACAGCAGCGATCCGTTGCATCC

TTGGGGGATCCTCTGCCACCCACGCCACAGTGGCAGCCGTAGAAGCCATAACTGAGTGCGGCTTCCTTTCCTGTCGTCAA

CTTGATCATTCTGTGGAAATTCACCAAATTCCCATGGGCCTGCAGTAGGCCAAAGATCATGATCACTGCCAACAGTAGGA

GGGTCTTCATGGCGATCGCGGCGGCAGATCTCCTCGGTACCGGATCCTCTCCCTATAGTGAGTCGTATTACTAGCGGATC

TGACGGTTCACTAAACCAGCTCTGCTTATATAGACCTCCCACCGTACACGCCTACCGCCCATTTGCGTCAATGGGGCGGA

GTTGTTACGACATTTTGGAAAGTCCCGTTGATTTTGGTGCCAAAACAAACTCCCATTGACGTCAATGGGGTGGAGACTTG

GAAATCCCCGTGAGTCAAACCGCTATCCACGCCCATTGATGTACTGCCAAAACCGCATCACCATGGTAATAGCGATGACT

AATACGTAGATGTACTGCCAAGTAGGAAAGTCCCATAAAGGTCATGTACTGGGCATAATGCCAGGCGGGCCATTTACCGT

CATTGACGTCAATAGGGGGCGTAC

Homo sapiens phospholipase A2, group IIA (platelets, synovial fluid) (PLA2G2A), transcript variant 4, mRNA

Sequence ID: ref|NM_001161729.1|Length: 940Number of Matches: 1

Related Information

Gene-associated gene details

GEO Profiles-microarray expression data

Map Viewer-aligned genomic context

Range 1: 217 to 650GenBankGraphics Next Match Previous Match

Alignment statistics for match #1

| Score | Expect | Identities | Gaps | Strand |
|---|---|---|---|---|
| 802 bits(434) | 0.0 | 434/434(100%) | 0/434(0%) | Plus/Minus |

Query 139 GCAACGAGGGGTGCTCCCTCTGCAGTGTTTATTGGAATAGTACTGGTACTTTTTATTGTA 198

||||||||||||||||||||||||||||||||||||||||||||||||||||||||||||

Sbjct 650 GCAACGAGGGGTGCTCCCTCTGCAGTGTTTATTGGAATAGTACTGGTACTTTTTATTGTA 591

Query 199 GGTCGTCTTGTTTCTAGCAAAACAGGTGGCAGCAGCCTTATCACACTCACACAGTTGACT 258

||||||||||||||||||||||||||||||||||||||||||||||||||||||||||||

Sbjct 590 GGTCGTCTTGTTTCTAGCAAAACAGGTGGCAGCAGCCTTATCACACTCACACAGTTGACT 531

Query 259 TCTGCAGGAGTCCTGTTTTGCACAGGTGATTCTGCTCCCCGAGTTGCTAAACTTGTAGCT 318

||||||||||||||||||||||||||||||||||||||||||||||||||||||||||||

Sbjct 530 TCTGCAGGAGTCCTGTTTTGCACAGGTGATTCTGCTCCCCGAGTTGCTAAACTTGTAGCT 471

Query 319 CAGAAATTTGGTGCCACATCCACGTTTCTCCAGACGTTTGTAGCAACAGTCATGAGTGAC 378

||||||||||||||||||||||||||||||||||||||||||||||||||||||||||||

Sbjct 470 CAGAAATTTGGTGCCACATCCACGTTTCTCCAGACGTTTGTAGCAACAGTCATGAGTGAC 411

Query 379 ACAGCAGCGATCCGTTGCATCCTTGGGGGATCCTCTGCCACCCACGCCACAGTGGCAGCC 438

||||||||||||||||||||||||||||||||||||||||||||||||||||||||||||

Sbjct 410 ACAGCAGCGATCCGTTGCATCCTTGGGGGATCCTCTGCCACCCACGCCACAGTGGCAGCC 351

Query 439 GTAGAAGCCATAACTGAGTGCGGCTTCCTTTCCTGTCGTCAACTTGATCATTCTGTGGAA 498

||||||||||||||||||||||||||||||||||||||||||||||||||||||||||||

Sbjct 350 GTAGAAGCCATAACTGAGTGCGGCTTCCTTTCCTGTCGTCAACTTGATCATTCTGTGGAA 291

Query 499 ATTCACCAAATTCCCATGGGCCTGCAGTAGGCCAAAGATCATGATCACTGCCAACAGTAG 558

||||||||||||||||||||||||||||||||||||||||||||||||||||||||||||

Sbjct 290 ATTCACCAAATTCCCATGGGCCTGCAGTAGGCCAAAGATCATGATCACTGCCAACAGTAG 231

Query 559 GAGGGTCTTCATGG 572

||||||||||||||

Sbjct 230 GAGGGTCTTCATGG 217

**Supplementary Figure 9: The Basic Local Alignment Search Tool (BLAST) of the sequencing results of pENTER-*tcf4* and pENTER-pla2g2a with NCBI database.** (**A**) 5′ sequencing results of pENTER-*tcf4* and its similarity to a sequence from NCBI database; (**B**) 3′ sequencing results of pENTER-*tcf4* and its similarity to a sequence from NCBI database; (**C**) 5′ sequencing results of pENTER-pla2g2a and its similarity to a sequence from NCBI database; (**D**) 3′ sequencing results of pENTER-pla2g2a and its similarity to a sequence from NCBI database

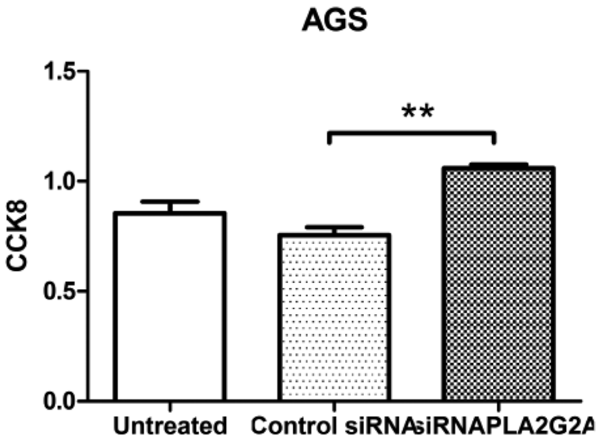


**Supplementary Figure 10: Cell counting kit 8 assay of sPLA2-IIA and control siRNA treated cells.** Experiments were performed in triplicate with similar results.
